# Nanoplasmonic Upconverting Nanoparticles as Orientation Sensors for Single Particle Microscopy

**DOI:** 10.1038/s41598-017-00869-3

**Published:** 2017-04-10

**Authors:** Kory K. Green, Janina Wirth, Shuang F. Lim

**Affiliations:** Department of Physics, North Carolina State University, Raleigh, NC 27695 USA

## Abstract

We showed that the anisotropic disk shape of nanoplasmonic upconverting nanoparticles (NP-UCNPs) creates changes in fluorescence intensity during rotational motion. We determined the orientation by a three-fold change in fluorescence intensity. We further found that the luminescence intensity was strongly dependent on the particle orientation and on polarization of the excitation light. The luminescence intensity showed a three-fold difference between flat and on-edge orientations. The intensity also varied sinusoidally with the polarization of the incident light, with an I_max_/I_min_ ratio of up to 2.02. Both the orientation dependence and I_max_/I_min_ are dependent on the presence of a gold shell on the UCNP. Because the fluorescence depends on the NP’s orientation, the rotational motion of biomolecules coupled to the NP can be detected. Finally, we tracked the real-time rotational motion of a single NP-UCNP in solution between slide and coverslip with diffusivity up to 10^−2^ μm^2^s^−1^.

## Introduction

In single biomolecule research, the dynamics and mechanisms of proteins and motors are often explained by monitoring their translations and orientations. Two commonly used tools for tracking protein orientations are rod-like Quantum dots (Qrods) and gold nanorods (AuNRs), but both approaches have limitations. Qrods were successfully used to simultaneously track the 3D orientation and movements of myosin V with 10° accuracy at 33 ms time resolution^1^. However, the random blinking frequency of Qdots^2^ coupled with the stochastic motion of the biomolecules being probed make data interpretation difficult. The AuNR shape gives rise to surface plasmon resonances followed by transverse (TSPR) and longitudinal localized surface plasmon (LSPR) modes; the latter mode is polarized parallel to the long axis of the AuNR^3^. Anisotropic absorption and scattering have also been correlated to the 2D orientation of the AuNR^4^. Hence, AuNRs were used to observe the rotations of *Escherichia coli* F_1_-ATPase^5, 6^ and microtubules on a kinesin-coated surface^7^. However, interpretation of the scattering signals is difficult due to the nanorods’ anisotropy. As the AuNRs rotate from 0° to 90°, considerable effort is needed to deduce the actual orientation of the AuNR from the variable scattering intensities that result from orientation between the two axes.

Recent studies have shown that rare earth upconverting nanoparticles (UCNP) offer an attractive alternative method for tracking orientation due to their inherent excitation polarization dependence^8^. UCNPs are excited in the near infrared and fluoresce via anti-Stokes emission in the visible energy range (400–800 nm), making them attractive for use in biological settings due to their low-energy excitation photon energy. UCNPs are excellent fluorescent probes, as they have no blinking (intermittent fluorescence), bleaching (reduced fluorescence over time), or fluorescence background due to their near infrared excitation. These properties set them apart from dyes and Qdots^9^, and allow dynamic molecular interactions to be tracked continuously in real time.

However, UCNPs are not as efficient as traditional Stokes dyes; therefore, several studies have tried to enhance the Anti-Stokes emission by coupling gold and silver NPs and shells to rare earth ion doped upconverting cores (NP-UCNP)^10–14^ in order to increase overall emission intensity and modify emission color^8, 10, 11, 13–16^. In general, two different types of plasmonic structures are used for enhanced upconversion, and their plasmon resonances overlap spectrally with either the excitation or the emission wavelength of the UCNP. Higher enhancements are achieved for nanostructures exhibiting resonances matching the UCNP’s absorption with different nanostructure types, e.g. pillar arrays^12, 17^, hole arrays^18^, and core–shell particles^11^. Further investigations have provided direct evidence that coupling individual plasmonic nanostructures^19, 20^ or coating with noble metals^21, 22^ can produce plasmonic-enhanced upconversion luminescence at the single particle level. The enhancement is attributed to coincidence of surface plasmon resonances and core energies.

Earlier we found that nanoplasmonic particles are considerably brighter, up to five fold increase, compared with unmodified UCNPs^8, 21^. In our time-resolved studies, we observed that the coupling between excited states and emitted light is profoundly altered by the presence of the metal, leading to the enhancement^8, 21^. Depending on the orientation of the nanodisk, the emission is also polarized and directional^23^, lending further complexity and richness to the photophysics of UCNPs. According to Chen *et al*., the fluorescence intensity of an on-edge oriented UCNP nanodisk shows a sinusoidal dependence on excitation polarization^24, 25^. They attributed this effect to the variation of the dipole oscillator strength spatially within the nanodisk due to the anisotropic nature of the surrounding crystal lattice. Herein, we confirmed the same effect by using spectral and time-resolved measurements.

**Figure 1:**
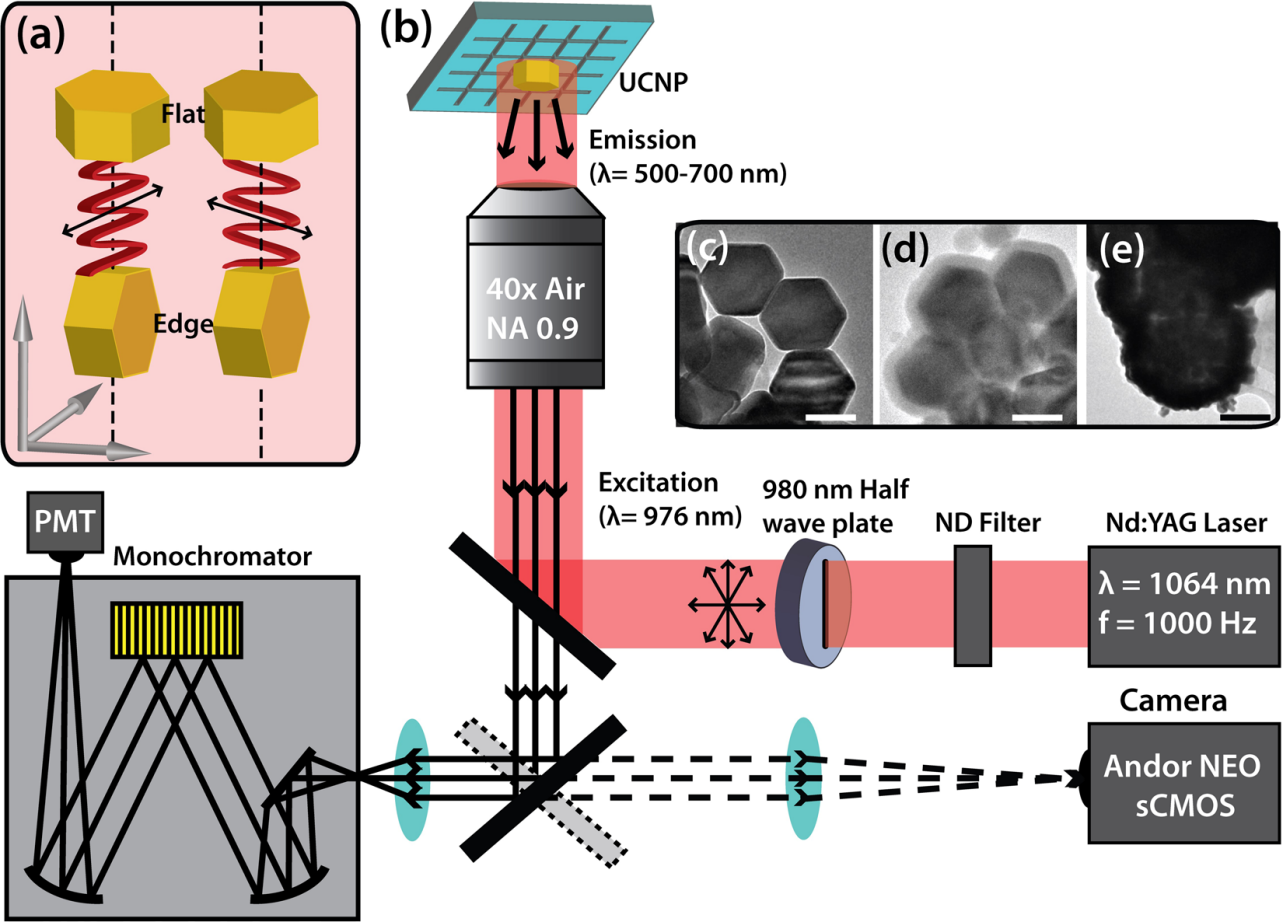
(**a**) Incoming polarization with respect to the orientation of either flat or on-edge particles. The optical experimental set up is displayed in (**b**). The modification process from unmodified UCNP (**c**) through silanization (**d**) to gold shell growth (**e**) is also shown.

In addition, anisotropically shaped NP-UCNPs oriented with flat configurations showed stronger emission intensity than those with edge orientations. Flat and edge orientations resulted in different decay rates (flat and edge UCNP orientations are shown in Fig. 1a).

Our experimental observations were supported by finite element calculations, and showed that the fluorescence orientation dependence is, in turn, dependent on the geometry of the NP-UCNP. Our current experimental observations correlate well with our earlier calculations on similar nanoplasmonic upconverting NPs with different orientations to the incident excitation and excitation polarization^8^.

In our experiments, we examined two separate populations of NP-UCNPs to investigate the effect of gold shell coupling on excitation polarization and orientation dependence. The NP-UCNPs with larger shape anisotropy (more disk-like shape) showed a larger distinction of the emission intensity with orientation, where a 3.5 times intensity difference was found between flat and edge orientation in the red spectral range from 640–680 nm, measured as area under the spectral curve. We also compared the intensity difference with more prism-shaped NP-UCNPs, where the emission intensity variation due to orientation was about 2.1 times for the same spectral range. For the latter, additional measurements of the single UCNP, oriented on edge, showed an excitation polarization dependence, where the I_max_/I_min_, ratio, defined as an on/off ratio between the highest emission intensity and lowest emission intensity, was obtained for a silica coated UCNP (I_max_/I_min_ = 1.72) and a NP-UCNP (I_max_/I_min_ = 2.02). In addition the more prism-shaped NP-UCNPs showed increased emission intensity due to gold shell coupling, facilitating their fluorescent tracking.

In this paper, we analyzed the diffusional characteristics of a single NP-UCNP tumbling in solution between coverslip and slide to confirm that the orientation dependent fluorescence of the single NP-UCNP can be used to track single molecules. The tumbling NP-UCNP clearly showed a bright/dark fluorescence and we performed autocorrelation of the ﻿fluorescent intensity to derive the diffusion constant. This orientation dependence of the emission intensity can be applied to track the 3D orientation of conjugated proteins.

## Results

### Immobilized Particle Orientation Dependence

Before single particle spectra were collected, the immobilized sample was inspected for isolated single particles. Figure S1 shows AFM confirmation of single particles from the population, showing edge versus flat orientation. After the fluorescent object was confirmed to be a single particle, fluorescence was collected using the system detailed in Fig. 1(b). By rotating the wave plate in the excitation beam path, the desired polarization with respect to the orientation of the UCNP, as determined by AFM imaging, was acquired.

**Figure 2:**
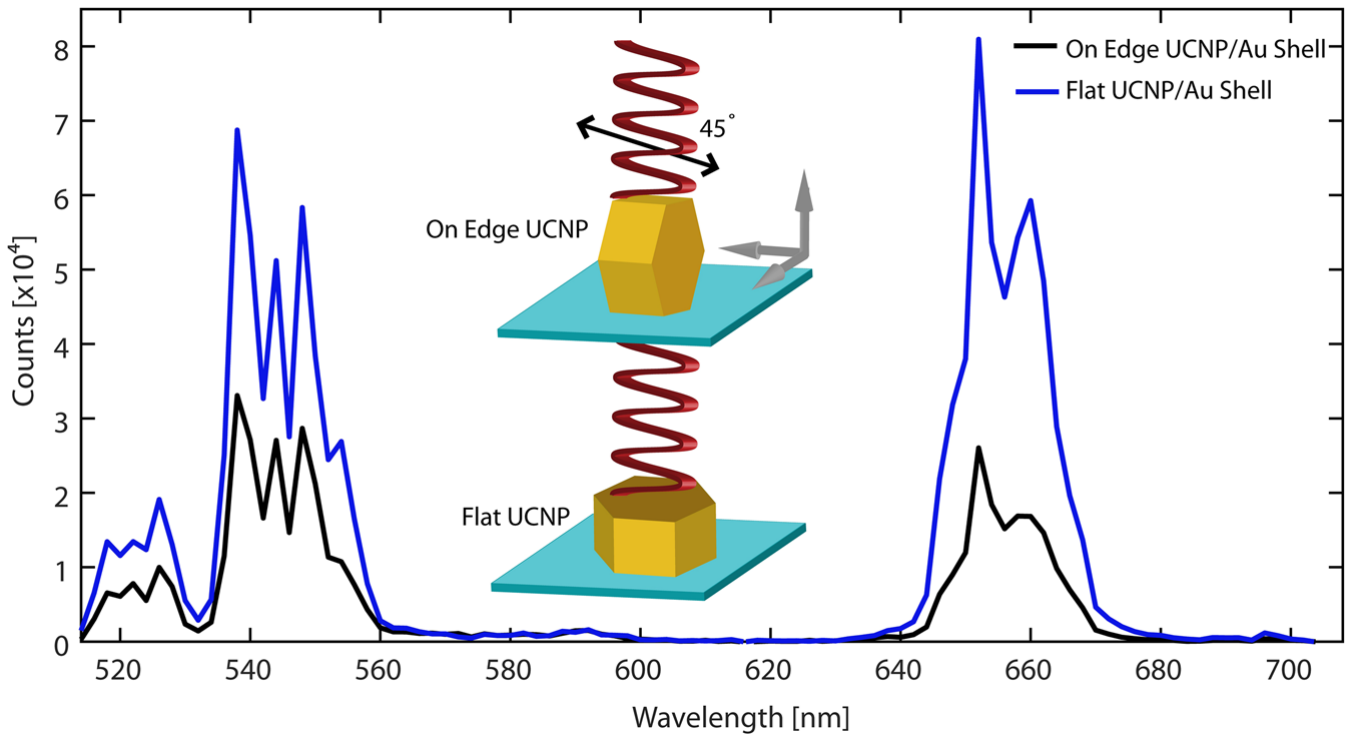
Single particle orientation dependent fluorescence spectra comparing UCNP lying flat (blue) and on edge (black). A 3- to 4-fold difference in intensity is observed at 640–680 nm. The spectral measurement standard deviation is less than 0.0018.

Figure 2 shows that the fluorescence intensity was strongly influenced by the orientation of the disk shaped NP-UCNPs (314.8 ± 26.9 nm in diameter, 182.9 ± 25.8 nm in height, coated with a 14.1 ± 7.2 nm thick gold shell). We observed an average 3- to 4-fold intensity difference when the orientation changed from edge to flat configuration. To understand how the orientation influences the emission intensity, we performed finite element calculations (see Supporting Info, S3).

**Figure 3:**
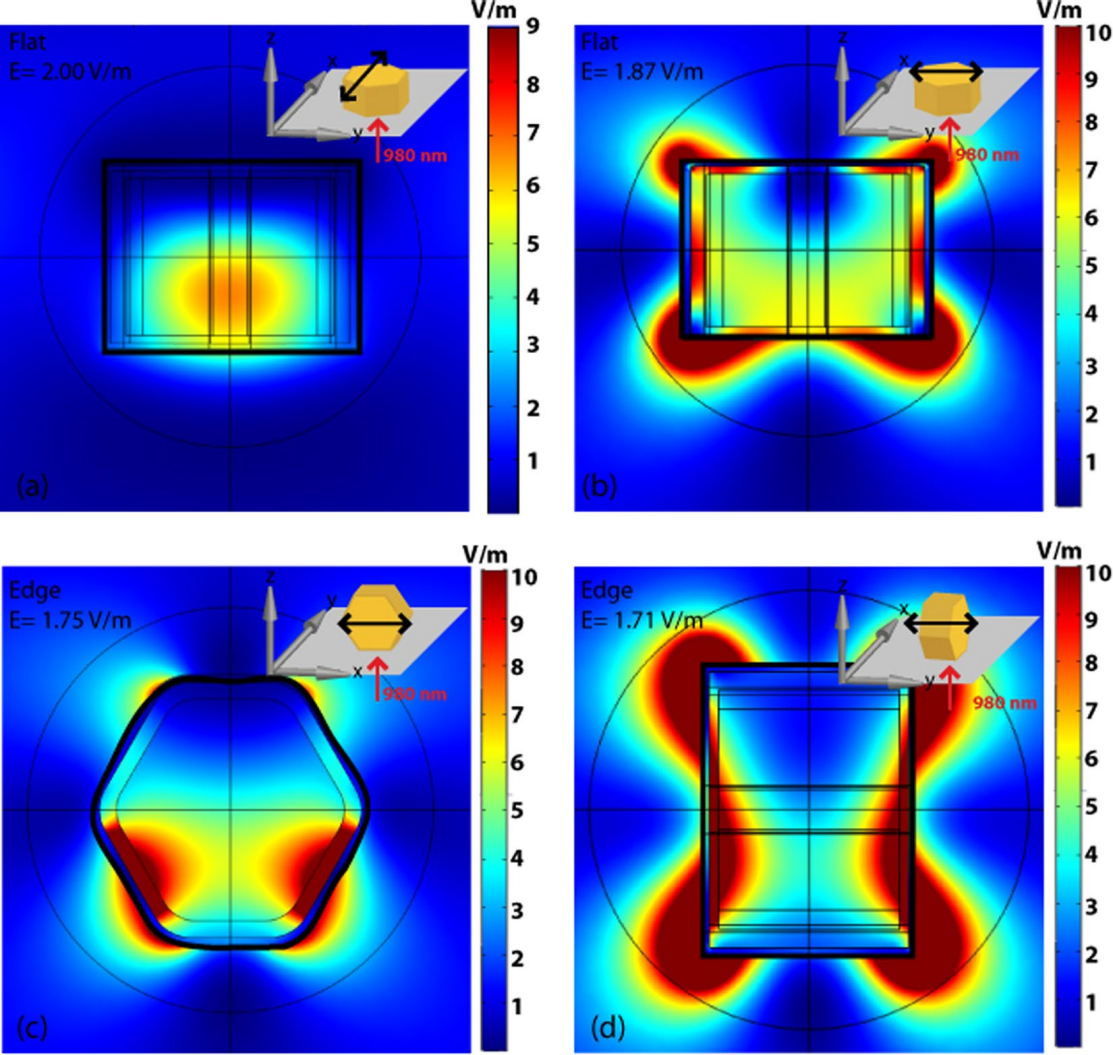
Plots of the volume averaged square of the electric field strength of flat (**a** and **b**) and edge orientation (**c** and **d**) at 0° (x-polarized, **a** and **c**) and 90° (y-polarized, **b** and **d**) excitation polarization. The direction of the incident light is shown by the red arrow. The orientation of the polarization is shown by the black arrow.

Our calculations used an exact model of the NP-UCNP and were performed with a plane wave excitation normal to the hexagonal face or normal to the particle oriented on its edge (Fig. 3). For the particle oriented ‘flat’ to the incident excitation, the volume averaged electric field strengths were 2.0 V/m (0° excitation polarization) and 1.87 V/m (90° excitation polarization) (Fig. 3a and b, respectively). For incident excitation of a particle oriented on its edge, the volume averaged electric fields were 1.75 V/m (polarization along the longer axis, 0 or 180°) and 1.71 V/m (polarization along the shorter axis, 90°) (Fig. 3c and d, respectively). In general, emission intensity scales with E^2n^, where n is the power exponent, which is typically 2 for a 2-photon upconversion process. Hence, higher electric field strength results in a large difference in emission intensity between the flat and edge orientations. Our calculations also showed that this distinction becomes larger for the more anisotropically shaped or disk-shaped NP (not shown). We believe that both our experimental and calculated results, as shown in Figs 2 and 3, respectively, show that the changes in optical properties are due to the change in the surface plasmon resonance with the orientation of the NP to the incoming excitation. According to our calculations in Fig. 3, the electric field strength within the core of the NP is higher for a flat orientation compared with that for an edge orientation.

**Figure 4:**
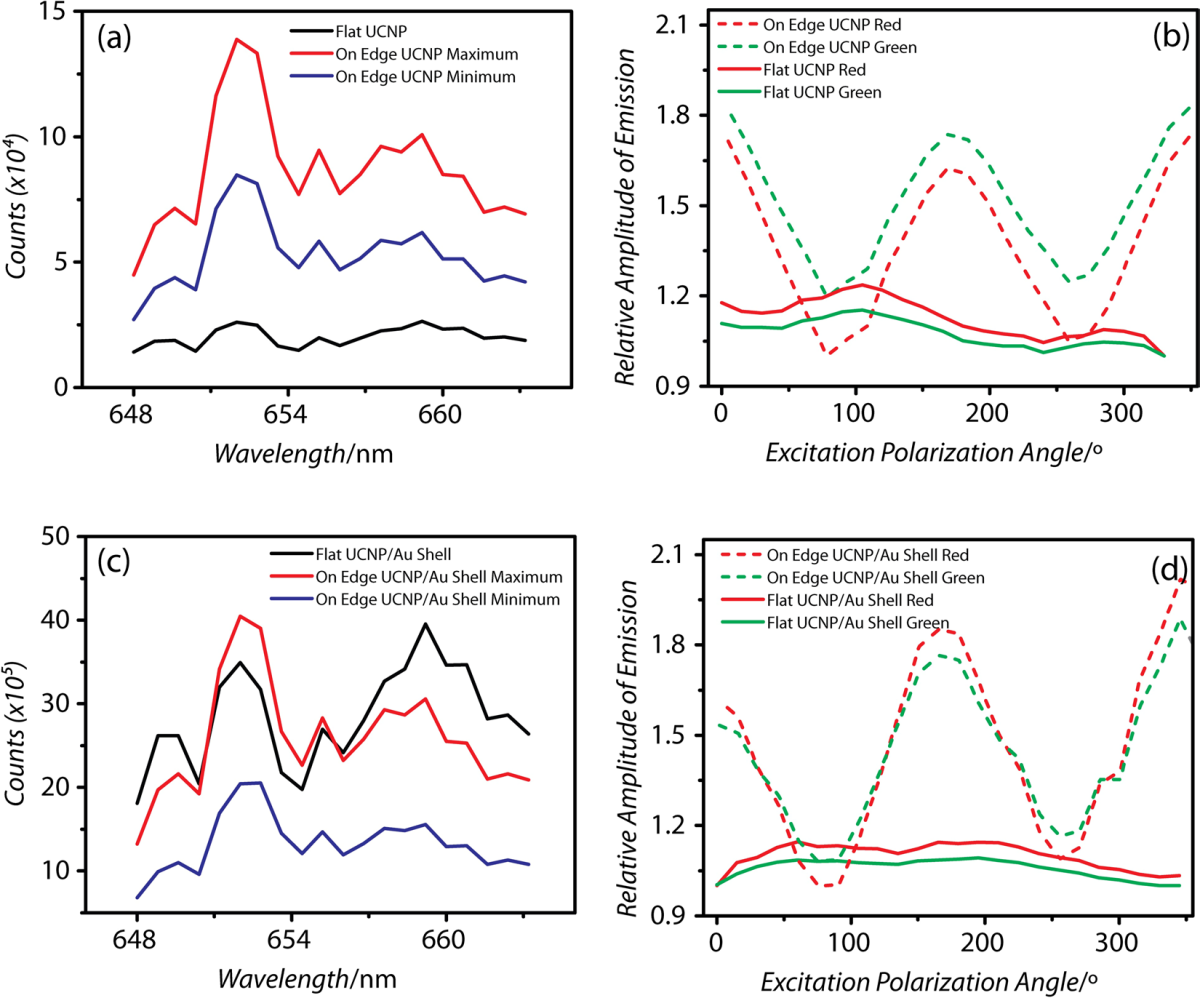
Red spectra (λ = 650 nm) of (**a**) unmodified UCNP, (**c**) UCNP with 18 nm Au Shell, and the emission intensity dependence on the excitation polarization for (**b**) unmodified UCNP and (**d**) UCNP with an 18 nm Au shell plotted as a ratio to the lowest emission intensity polarization angle (I_angle_/I_min_). Excitation intensity is 1.68e3 W/cm^2^ and spectral measurement standard deviation is less than 0.001.

In addition to the flat versus edge orientation dependence, Fig. 4 shows spectra taken from a smaller, less anisotropic set of UCNPs (152.6 ± 8.3 nm in diameter, 121.2 ± 4.2 nm in height) with and without 18.1 ± 4.3 nm thick gold shell. As seen in Fig. 4(a) and (c), the differences between flat and edge orientations were much less pronounced than those with the larger more anisotropic UCNP. In the NP-UCNPs, the flat particle emission was typically larger in intensity than the edge oriented particle emission at 90° excitation polarization.

In Fig. 4(a) and (c), a comparison of flat unmodified UCNP (Fig. 4a, black line, area under curve) and flat NP-UCNP (Fig. 4c, black line, area under curve) shows an average difference in emission intensity of around 4.9 times in the red and 5.2 times in the green. Edge NP-UCNPs showed 2.6 times enhancement in the red and 2.1 times enhancement in the green. These data were consistent with our earlier observations, where the surface plasmon resonance of the gold shell led to greater emission of the rare earth dopants^8, 21^. The slightly larger enhancement noted here, compared with that in our prior work, is attributed to the optimized geometry of the UCNP core diameter and gold shell thickness, coupled with measurements at the single particle level, where ensemble effects (aggregates, defective gold shells) are eliminated.

**Figure 5:**
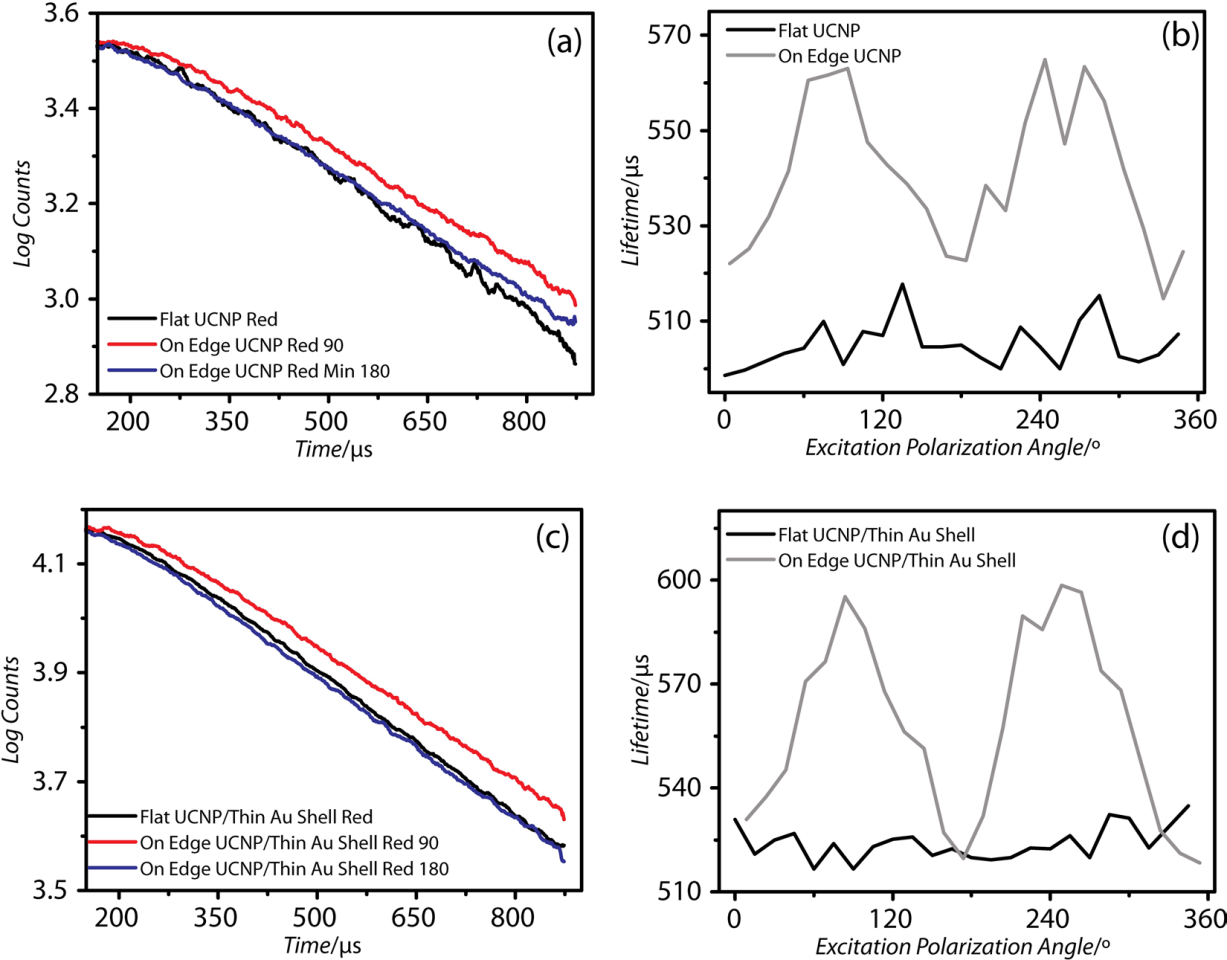
Time-resolved decay of (**a**) unmodified UCNP and with (**c**) 18 nm gold shell coating (NP-UCNP) with corresponding dependence of the radiative lifetime on the excitation polarization angle for the Er^3+^; ^4^F_9/2_ to ^4^I_15/2_ transition for unmodified (**b**) and NP-UCNP (**d**). The spectral measurement standard deviation is less than 0.001.

Furthermore, both the unmodified and NP-UCNP displayed similar behavior in their polarization dependence. Figure 4 (b) and (d) indicate the intensity contrast between flat and edge orientations and the effect of the excitation polarization direction. With flat oriented particles, the emission intensity was not dependent on the excitation polarization. With the edge oriented particles, the emission intensity of both unmodified and gold shell coated UCNPs showed strong sinusoidal dependence on the excitation polarization. The on/off ratio of measured I_180_/I_90_ was 1.72 for bare UCNP and 2.02 for NP-UCNP. We attribute the polarization dependence on the preferred, non-uniform dipole orientations along certain axes within the anisotropic nanocrystal, as observed by Zhou *et al*.^26^. In addition, in agreement with Chen *et al*., we found no polarization dependence of the excitation emission along the nanocrystal’s optical axis, which is normal to the hexagonal face^23^. Our time-resolved measurements shown in Fig. 5 and finite element calculations, shown later, support our observations. Thus, the gold shell does not modify the polarization dependence of the UCNP emission.

In addition to the spectral intensity measurements, time resolved data were collected simultaneously for each wavelength. Figure 5 shows the corresponding time-resolved decay of the same unmodified UCNP and NP-UCNP as shown in Fig. 4. As shown in Fig. 5(a) and (c), both flat and edge configurations exhibited mono-exponential radiative decay.

Distinctly different decay rates were observed for both orientations. The edge orientation at 90° excitation polarization displayed a much slower rate of fluorescence decay compared with the edge orientations at 0° and 180° or with the flat orientation. We calculated the electric field profile within the UCNP core (see Supporting data, Fig. S2) and found a steep drop in the electric field strength for the flat orientation upon initial excitation on the front face of the nanocrystal. The electric field strength decreased from 2.4 to 0.1 V/m (0° polarization) and from 2.5 to 0.4 V/m (90° polarization) (see Fig. S2a). A similar decrease from 2.0 to 0.6 V/m occurred for the edge orientation at 0° excitation polarization (see Fig. S2b). However, a much lower drop in the electric field strength from 1.6 to 1.2 V/m was found for the edge orientation at 90° excitation polarization (see Fig. S2b). In general, for flat (0° and 90° excitation polarization) and edge orientations (0° excitation polarization), the incoming excitation decreased in intensity as it traversed the nanocrystal core, suggesting that emission takes place largely on one side of the nanocrystal. In contrast, in edge oriented particles at 90° excitation polarization, the electric field was localized on both the front and back surfaces of the nanocrystal (see Fig. 3d and Fig. S2b). This difference may have contributed to the lower decay rate seen in Fig. 5c for edge oriented particles at 90°. The same explanation could be applied to the dip in the emission intensity at 90° excitation polarization (Fig. 4d). Hence, the electric field distribution within the UCNP core is strongly influenced by the particle orientation and the polarization of the incoming excitation. We further speculate that this effect occurs independently of the presence of a gold shell, since in Fig. 5a, the unmodified UCNPs show a similar behavior in decay rate.

Figure 5 (b) and (d) show the lifetime derived from the mono-exponential fit of the fluorescence decay measured at different excitation polarizations. Our results show that, similarly to the emission intensity dependence, only the edge orientation results in a sinusoidal dependence of the lifetime with excitation polarization. Therefore, orientation could be tracked in both emission intensity and emission lifetime of these states.

**Figure 6:**
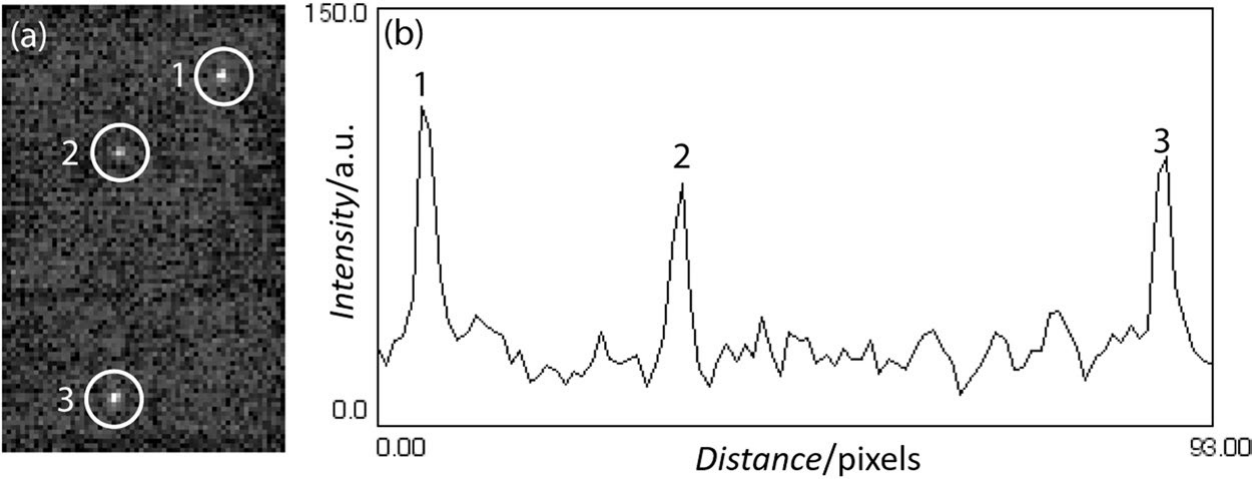
(**a**) Wide field fluorescence of one selected frame (frame no. 282) from the movie (see Supporting, M1. UCNP in sucrose) with NP-UCNPs in 50% sucrose showing 3 particles (1 to 3). (**b**) Corresponding positional time trace of the selected particles. Particles 1 and 2 were considered for fluorescence intensity autocorrelation analysis.

To demonstrate the bright/dark change in intensity with rotation of the disk shaped NP-UCNP, a 10 µg/ml concentration of 315 nm diameter UCNP coated with a 11 nm thick gold shell was suspended in 50% sucrose solution (V = 20 µl) between a slide and coverslip. We observed a bright to dark change in intensity when the NP-UCNPs tumbled in solution and recorded a movie of the process by using the same setup as shown in Fig. 1b with a frame rate of 10 Hz (see Supporting Data, Movie). Figure 6(a) shows a single movie frame of the wide field fluorescence of particles marked 1 to 3, and Fig. 6(b) shows their corresponding positional time trace.

**Figure 7:**
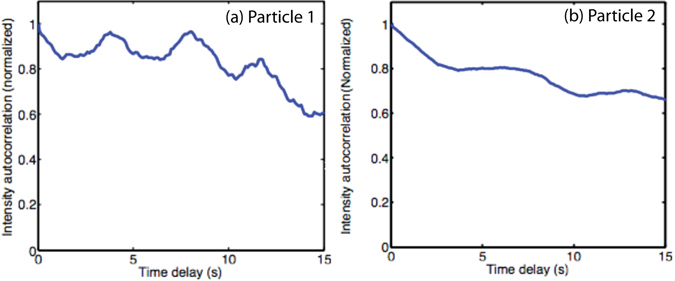
Intensity autocorrelogram for (**a**) particle 1, and (**b**) particle 2. Comparing rotational (**a**) and translational (**b**) motion.

We performed an autocorrelation of the intensity to distinguish between rotational and diffusional motion of the NP and calculated the mean square translational displacement to derive the diffusional constant. Figure 7 shows that particle 1 underwent periodic tumbling superimposed on random diffusion. In general, random diffusion is described by an exponential function. In comparison, particle 2 showed less periodic tumbling, but more random diffusion, as shown by the shape of the autocorrelation curve. From both particles 1 and 2, we estimated a translational diffusional constant of about 10^−2^ μm^2^ s^−1^, which is comparable with typical diffusion constants of Qdot labeled single molecule proteins^27, 28^.

## Conclusion

In conclusion, we found that the shape asymmetry of the UCNP itself contributes strongly to the orientation and excitation polarization dependence of the emission intensity. The presence of a gold shell enhances the intensity contrast between flat and edge orientations. Potential applications, such as single particle rotational tracking, are envisioned. As a proof of concept, we analyzed a particle tumbling in solution to show that the diffusional constant of a single particle can be determined. Further investigation with a larger sample number is underway to quantify both the rotational and diffusional constants of NP diffusion.

## Methods

### Sample preparation

NaYF_4_: Yb, Er NPs with a molar concentration of 20% Yb^3+^ and 2% Er^3+^ were synthesized in the manner described in our previous work^29^. After washing and drying, UCNPs were dispersed in cyclohexane to coat them with a silica shell. Coated UCNPs were then functionalized with amine groups by silanization with amino propyltriethoxysilane (APTES). Negatively charged gold seeds were then nucleated in solution with the silica coated UCNPs. After attachment, gold growth was facilitated by the reduction of additional gold salt allowing the gold seeds to grow, coalesce, and form a complete shell. To control the shell’s thickness, different concentrations of gold salt were added to the growth medium. To stop the growth process and prevent aggregation, thioglycolic acid was added to the growth medium after 30 minutes of reaction time. TEM imaging performed on a JEOL 2000FX TEM (NCSU AIF) was used to characterize the UCNPs and resulting composite structures (see Supporting data, Fig. S4). TEM grids were prepared by drop casting 10 µl of (1 µg/ml) UCNP/Au-Shell solutions.

To perform correlated structural and optical single NP spectroscopy, we used quartz glass chips with a chromium grid pattern, sized for correlated bright field, fluorescence, and AFM imaging. The chips were fabricated by lift-off process in the cleanroom at the NCSU Nanofabrication Facility (NNF).

Aqueous solutions (V = 50 µl) of NP-UCNPs (1 µg/ml) and SiO_2_@UCNPs (100 ng/µl) were pipetted on the chips and incubated for 30 min at RT while shaking at 300 rpm, followed by washing in 5 ml DI water for 5 min and drying under nitrogen flow. For better attachment of the NP-UCNPs, the glass chips were functionalized with thiol-terminated silane layers prior to immobilization. Therefore, the chips were first cleaned by an oxygen plasma (300 W, 5 min), incubated overnight in mercaptopropyltriethoxysilane (MPTES, 300 mM in toluene) at 75 °C, washed for 10 min in toluene in an ultrasonic bath, and finally dried under nitrogen and stored for 1 h at 90 °C.

### Correlative microscopy

For optical correlation, a wide field fluorescence image of the UCNPs was taken and their positions recorded on the indexed grids with an Andor NEO sCMOS camera. Afterwards, the recorded positions were characterized with a MFP-3D-BIO Atomic Force Microscope (AFM, Asylum Research) in tapping mode to find single UCNPs with different orientations, either lying with flat or edge orientations (see Supporting, Fig. S1).

### Time resolved spectroscopy measurements

All spectroscopic studies were performed on single particles as identified by the above section using an optical microscope (40x, 0.9 N.A. air objective) utilizing the same imaging camera to locate the desired particles. Spectral properties were measured using a half-meter monochromator coupled to a Hamamatsu H7421-40 photomultiplier. A tunable Optical parametric oscillator (OPO) was pumped by a neodymium-doped yttrium aluminum garnet (Nd:YAG) laser to facilitate excitation at 980 nm (1000 Hz, 4.5 ns pulse width). Acquisition of the photomultiplier was timed with the pulsed emission to resolve the time-dependent behavior at each wavelength. For polarization dependence, a ThorLabs WPH05M-980 wave plate was inserted into the excitation path and rotated to provide excitation polarization in the desired angles (see Fig. 1).

## Electronic supplementary material

Movie 1

Supplementary Information
