# Supplementary material for: Nanoplasmonic Upconverting Nanoparticles as Orientation Sensors for Single Particle Microscopy
Source: Sci Rep. 2017 Apr 10;7:762. doi: 10.1038/s41598-017-00869-3 (PMC5429696; doi:10.1038/s41598-017-00869-3)
Supplement: Supplementary file 2 — Supplementary Information [file 41598_2017_869_MOESM2_ESM.pdf]

Supporting Information

Nanoplasmonic Upconverting Nanoparticles as Orientation Sensors for Single Particle Microscopy

Kory K. Green[1], Janina Wirth[1], Shuang F. Lim[1,]*

[1] Department of Physics, North Carolina State University, Raleigh, NC 27695, USA
* sflim@ncsu.edu

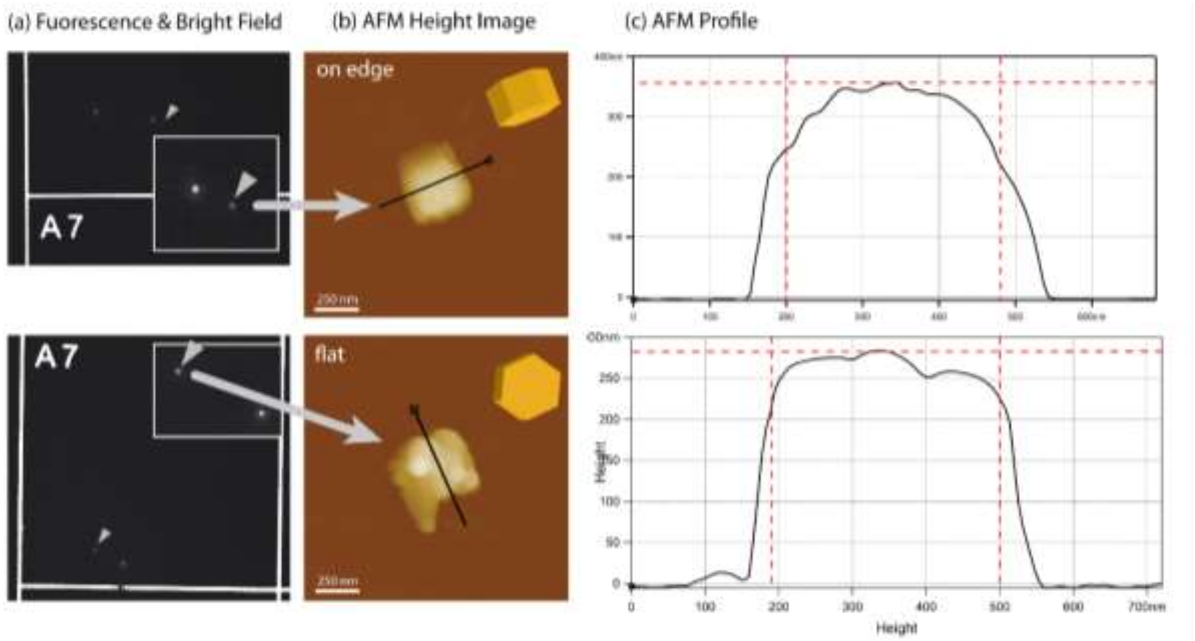

Figure S1. (a) Overlaid wide bright field and fluorescence image of one selected flat and edge oriented UCNP/Au-Shell. Inset shows a magnified image. (b) Corresponding AFM images of selected particles (grey arrows in (a)) with corresponding (c) AFM profiles.

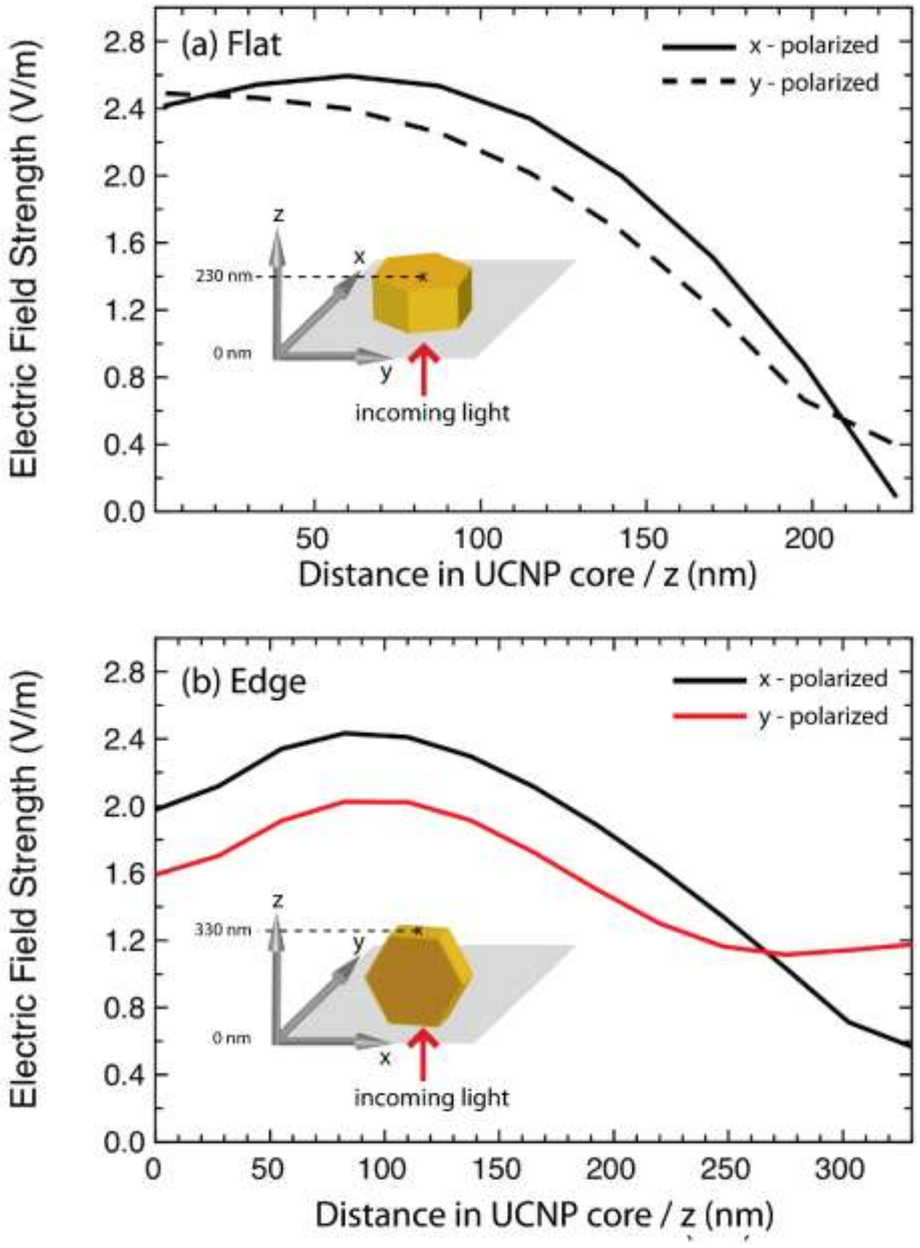

Figure S2. Electric field profile within the UCNP/Au-Shell nanocrystal core at x: 0 degrees, and y: 90 degrees, polarization for (a) flat, and (b) on edge, orientation.

(S3) Finite Element Calculation Procedure

COMSOL modelling was performed for UCNP with a 20 nm thick silica layer and an equivalent gold shell thickness of 11 nm.

We modeled a perfect continuous gold shell of lower thickness of about 8 nm, in place of a real gold discontinuous gold shell with voids. We used Brugemmans effective medium

theory [25] to calculate the thickness of a uniform gold shell that would accurately represent the imperfect experimental gold shell thickness. We calculate the expected measured thickness t_{exp} by requiring the same propagation loss for light passing through the perfect model film t_{Au} as through the imperfect film by setting the Beer's Law exponents to be the same, $n_{i,Au}\ t_{Au} = n_{i,eff}\ t_{exp}$, with gold dielectric constant at 960 nm [26], $(n_{r,Au}+n_{i,Au})^2 = -46.797 + 3.0\ i$. In order to obtain the effective index of the real film, $n_{r,eff}+n_{i,eff}$, we use the gold dielectric constant, the silica layer index of refraction of 1.52, and the gold volume fraction estimated from the transmission electron micrographs to be 0.6. The calculated result for the expected measured thickness t_{exp} = 14.0 nm, which is in reasonable agreement with the measured 14 nm given the uncertainty in the volume fraction measurement.

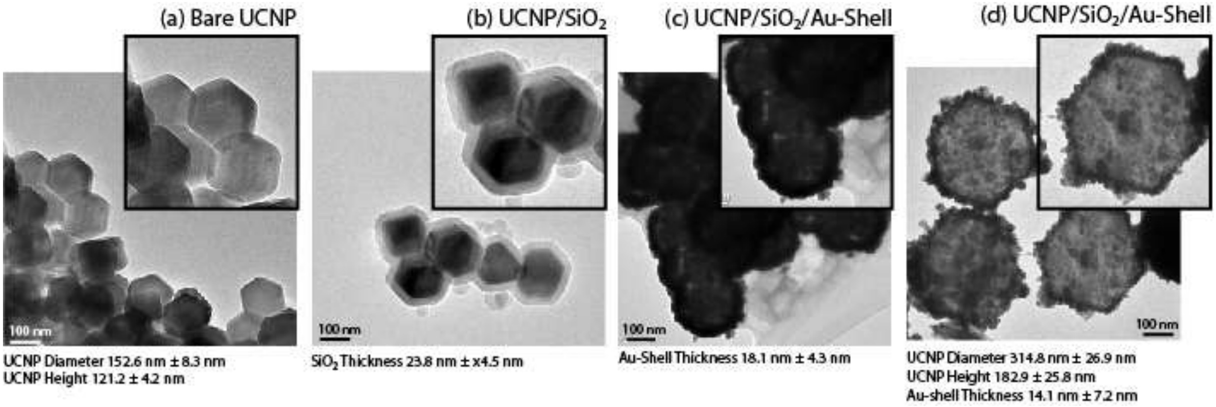

Figure S4. TEM images of (a) 152 nm bare UCNPs, (b) 152 nm UCNPs with 24 nm SiO_2-Shell, (c) 152 nm UCNPs with 24 nm thick SiO_2-Shell and 18 nm thick Au-Shell. (d) 315 nm UCNPs with 24 nm thick SiO_2-Shell and 14 nm thick Au-Shell. Insets show a more detailed view of the UCNPs.

34. D. E. Aspnes, Local-field effects and effective-medium theory: A microscopic perspective, *American Journal of Physics* 50, 704 (1982).
35. P. B. Johnson, R. W. Christy, Optical Constants of the Noble Metals *Physical Review* B **6**, 4370 (1972).

(Mov1) The movie is collected at 10 Hz. Each pixel represents 0.1625 um. NP-UCNPs in a 50% sucrose solution are free to diffuse between a slide and coverslip.
